# Association between living habits, indoor humidity, ventilation and asthma among residents in the tropical regions of China

**DOI:** 10.3389/fpubh.2023.1294115

**Published:** 2023-11-15

**Authors:** Mingming Chen, Kun Zhang, Xin Zhang, Jun Gao, Rongguang Zhang, Xiaoli Wei, Wenting Cao

**Affiliations:** ^1^Department of Epidemiology, International School of Public Health and One Health, Hainan Medical University, Haikou, China; ^2^Department of Geriatric Center, Hainan General Hospital, Haikou, China; ^3^School of Basic Medicine and Life Science, Hainan Medical University, Haikou, China

**Keywords:** asthma, damp and musty, living time, hair dyeing, education years

## Abstract

**Objective:**

Asthma is a major public health problem that affects both children and adults, and its prevalence varies among people with different climatic characteristics and living habits. However, few studies have investigated the prevalence and risk factors for asthma among tropical residents in China. Therefore, this study aims to investigate the correlation between individuals’ living environment and daily habits in Hainan Province, and the occurrence of asthma.

**Methods:**

This cross-sectional study collected data from 1021 participants in three regions of the Hainan Province. A questionnaire derived from the European Community Respiratory Health Survey was used to collect data on demographics, living habits, self-reported asthma, and respiratory system-related symptoms. Logistic regression was used for univariate and multivariate analyses to screen for relative risk factors associated with asthma.

**Results:**

Among the 1,021 subjects investigated, the prevalence rate of self-reported asthma was 18.6%. Significant risk factors for asthma include hair dyeing, longer living time in Hainan, higher BMI, and living in a damp and musty room. Protective factors included fruit intake, years of higher education, and indoor timing of natural ventilation.

**Conclusion:**

Higher frequency of hair dyeing, higher body mass index (BMI), longer living in Hainan, lower frequency of fruit intake, fewer years of education, a damp and musty room, and no indoor timing natural ventilation were associated with an increased risk of asthma.

## 1. Introduction

Asthma is a prevalent chronic, non-communicable disease that affected approximately 272.68 million people globally in 2017 (1). It is a prevalent respiratory disease characterized by airflow obstruction, airway inflammation, and airway hyperresponsiveness. These symptoms lead to wheezing, coughing, chest tightness, and shortness of breath in patients (2). This is a significant contributor to disability, health resource usage, and a diminished quality of life worldwide. Treatment of asthma with leukotriene receptor antagonists, either as monotherapy or in combination with inhaled corticosteroids (ICS), can effectively reduce asthma exacerbation. Additionally, biotherapy and macrolide antibiotics have emerged as potential treatment options in recent years (3).

The estimated global prevalence of doctor-diagnosed asthma in adults was 4.3% (95%CI: 4.2 ~ 4.4). However, there are significant variations between countries, with the highest prevalence rate found in developed countries such as Australia (21.5%) and the lowest in developing countries such as Vietnam (1.0%) (4). Using standardized data obtained from the World Health Organization (WHO) World Health Survey (WHS), we estimated that the global prevalence of clinical asthma in adults was 4.5% (4). A national cross-sectional study showed that the overall prevalence rate of asthma in China was 4.2% (5), implying a low diagnostic rate of asthma in the Chinese population and probably underestimating the true prevalence of the disease. Asthma is a complex condition characterized by variable respiratory symptoms and airflow limitations. Asthma exacerbations are typically associated with exposure to environmental allergens or upper respiratory tract infections, such as viruses (6). Previous studies have identified several risk factors for asthma, including age, exposure to mold, living in damp housing, smoking, exposure to mite mix, and cockroach allergens (5, 7–10).

Although allergies have traditionally been associated with western countries, they are also prevalent in developing countries.

Recent reports indicate that in the Asia-Pacific region, the prevalence of allergic diseases has reached its highest level in the last 50 years (7), a trend also observed in the tropical region of Southeast Asia. Furthermore, research has shown that in tropical regions, poly sensitization and seasonal variations complicate the management of allergic diseases. The causative agents of allergy in the tropics differ significantly from those observed in temperate countries (11). For example, Allergenic fungi are frequently present in Asian countries located in the tropics, where high levels of humidity and warm climates provide favorable conditions for their growth (12). As a result, a large number of fungal spores are airborne (13). These environmental conditions can potentially impact asthma symptoms.

Hainan province is the only province in China located entirely in tropical areas, and it possesses unique climate characteristics and dietary habits. This study aimed to investigate the prevalence rate of self-reported asthma in Hainan Province and explore the association of living habits, indoor humidity, and ventilation with asthma. A comprehensive understanding of the interrelationship between the factors under study can assist in the development of targeted interventions aimed at improving lifestyle habits, reducing the occurrence of asthma, and enhancing the quality of life in Hainan, China.

## 2. Materials and methods

### 2.1. Study design

From April to August 2021, this cross-sectional study was conducted in three administrative districts with varying humidity in Hainan Province, including Haikou, Chengmai, and Dongfang City. The study utilized stratified cluster random sampling to select subjects more than 18 years old from two universities in Haikou and Chengmai City, with a small portion of ordinary residents in Dongfang City.

### 2.2. Data collection

Data were collected using questionnaires derived from the European Community Respiratory Health Survey (ECRHS). This study investigated the basic demographic characteristics of subjects, including age, sex, ethnicity, years of education, whether parents had asthma, living time in Hainan, and BMI. In addition, the questionnaire covered the dietary information of subjects, whether animals were raised, whether the room was damp and musty, and whether there was indoor timing of natural ventilation. “There were more than three wheezing episodes during the past any time and there was wheezing when breathing out” was used as the standard for self-reported asthma.

The clinical features of self-reported asthma patients were also investigated, such as whether there were repeated attacks (more than three times) in the past 12 months, how many wheezing attacks occurred in the past 12 months, how long the repeated attacks of suffocation and wheezing symptoms lasted in the past 12 months, and whether the most severe stages in the past included inability to sleep, walk, suffocation at night.

Before the survey began, the questionnaire was reviewed and all relevant staff participating in the survey received training to ensure consistency. All participants completed the questionnaire anonymously with informed consent. The study was approved by the Ethics Review Committee of Hainan Medical University.

### 2.3. Statistical analysis

Basic demographic information and clinical characteristics of self-reported asthma patients were described using frequency and percentage. The prevalence rate of self-reported asthma was displayed according to the characteristics of the different populations, and univariate analysis was performed using the chi-square test. The compared variables included sex, age, living time in Hainan Province, ethnicity, years of education, BMI, whether pets were kept in the home, whether there was damp and musty in the room, whether there was timing natural ventilation in the room, whether parents had asthma, fruit intake frequency, vegetable intake frequency, meat intake frequency, exercise frequency, hair dyeing frequency, smoking frequency, drinking frequency, and mattress cleaning frequency. Self-reported asthma was used as the dependent variable, and the statistically significant indicators in the univariate analysis were used as independent variables. Binary logistic regression conditional forward method was used to screen for risk factors. The multifactorial regression model used in this study considered a wide range of factors, including general demographic characteristics, parental history of asthma, living environment, and local dietary behaviors. By identifying the risk factors through this model, we can develop targeted asthma prevention and control measures that cater to the needs of tropical residents. Statistical analyses were performed using IBM SPSS Statistics version 26.0(IBM SPSS Inc., Chicago, IL, United States). A significance level of *p* < 0.05 was used for all analyses. Figures were conducted using R software version 4.0.3 (R Development Core Team, Vienna, Austria).

## 3. Results

### 3.1. Characteristics of the study subjects

A total of 1,033 subjects were included in this survey, and 1,021 (98.8%) subjects were included for further analysis. Among the participants investigated, 367 were male (35.9%) and 654 were female (64.1%). The majority of the participants (77.5%) were range from 18 to 25 years of age. The Han ethnicity accounted for the largest proportion (85.4%) of 872 subjects, followed by the Li ethnicity (55 subjects, 5.4%) and other ethnicities (94 subjects, 9.2%). The largest period of education was 9–13 years, with 800 participants (78.4%). A total of 329 participants (32.2%) had lived in Hainan for more than 20 years. A total of 67 people (6.6%) kept pets, 427 subjects (41.8%) lived in damp and musty rooms, and 676 subjects (66.2%) had indoor timing natural ventilation. Seven mothers (0.7%) and 12 fathers (1.2%) had asthma (Table 1).

**Table 1:** Basic characteristics of survey subjects.

Variable	Total number of people	Percentage (%)	Variable	Total number of people	Percentage (%)
Gender			Pets keeping in home		
Male	367	35.9	Yes	67	6.6
Female	654	64.1	No	954	93.4
Age (year)			The room was damp and musty		
<25	791	77.5	Yes	427	41.8
25 ~ 49.9	107	10.5	No	594	58.2
≥50	123	12.0	Indoor timing natural ventilation		
Ethnicity			Yes	676	66.2
Han	872	85.4	No	345	33.8
Li	55	5.4	Mother has asthma		
Other	94	9.2	Yes	7	0.7
Education years (year)			No	1,014	99.3
<6	98	9.6	Father has asthma		
6 ~ 9	85	8.3	Yes	12	1.2
9 ~ 13	800	78.4	No	1,009	98.8
≥13	38	3.7	Living time in Hainan (year)		

### 3.2. Basic clinical feature of asthma patients

A total of 186 out of 1,021 subjects self-reported asthma, with a prevalence rate of 18.2%. Among the self-reported asthma patients in this survey, 15.6% had repeated episodes in the last 12 months, 4.8% had breathlessness and wheezing lasting less than four weeks, and 12.9% had irregular symptoms that could occur in any season or within 12 months. The other clinical characteristics are shown in Table 2.

**Table 2:** Basic clinical feature of asthma patients.

Variable	Total number of people	Percentage (%)	Variable	Total number of people	Percentage (%)
Have you had episodes in the last 12 months (more than 3 times)			Impact of asthma on life at initial onset		
Yes	186	18.2	No impact	5	2.7
No	835	81.8	Mild	5	2.7
How many wheezing episodes have you had in the last 12 months?			Moderate	4	2.2
Never	9	4.8	Serious	1	0.5
1–3 times	4	2.2	In which season or month do you usually have episodes of wheezing and suffocation?		
4–12 times	6	3.2	Disorder	24	12.9
>12 times	2	1.1	Perennial	5	2.7
How long have you been experiencing these recurring breathlessness and wheezing symptoms in the last 12 months?			Spring	4	2.2
Within 4 weeks	9	4.8	Summer	4	2.2
1–3 months	3	1.6	Autumn	2	1.1
3–6 months	1	0.5	Winter	3	1.6
>6 months	2	1.1	January	8	4.3
In the past when the symptoms were the most severe, have you experienced the following conditions due to wheezing, suffocation and chest tightness?			February	3	1.6
Unable to fall asleep in the supine position due to asthma attack	8	4.3	March	3	1.6
Unable to walk due to wheeze	6	3.2	April	6	3.2
Suffocated at night	9	4.8	May	4	2.2
Due to severe breathing cannot say the whole sentence	6	3.2	June	4	2.2
Suffocation due to acute attack of asthma is almost life-threatening	5	2.7	July	3	1.6
Effect of asthma on life in recent 12 months			August	5	2.7
No impact	5	2.7	September	5	2.7
Mild	7	3.8	October	2	1.1
Moderate	3	1.6	November	3	1.6
			December	6	3.2

### 3.3. Univariate analysis

The results of the univariate analysis showed that there were significant differences in different groups of sex, age, living time in Hainan, ethnicity, years of education, BMI, hair dyeing frequency, drinking frequency, fruit intake frequency, exercise frequency, mattress cleaning frequency, whether the room was damp and musty, and whether the room was timing natural ventilation (*p* < 0.05). The prevalence rate of self-reported asthma in male was higher than that in female (*p* < 0.05). The prevalence increased with age, living time in Hainan, and BMI (*p* < 0.05). Indoor humidity and a musty environment, hair dyeing, and drinking were risk factors (*p* < 0.05). In contrast, years of education, indoor timing of natural ventilation, higher fruit intake frequency, and exercise frequency appropriately decreased the prevalence rate of asthma (*p* < 0.05) (Table 3).

**Table 3:** Results of univariate analysis (*n* = 1,021).

Variable	No asthma *n* (%)	Have asthma *n* (%)	*p*	Variable	No asthma *n* (%)	Have asthma *n* (%)	*p*
Gender			0.040	Hair dyeing frequency			0.001
Male	288 (78.5)	79 (21.5)		Never	516 (97.5)	13 (2.5)	
Female	547 (83.6)	107 (16.4)		≤1 time/year	159 (98.8)	2 (1.2)	
Age (years)			<0.001	2 ~ 12 times/year	83 (95.4)	4 (4.6)	
18 ~ 24.9	753 (95.2)	38 (4.8)		1 ~ 3 times/month	15 (83.3)	3 (16.7)	
25 ~ 49.9	49 (45.8)	58 (54.2)		1 ~ 6 times/week	4 (80.0)	1 (20.0)	
50 ~ 90	33 (26.8)	90 (73.2)		≥1 time/day	9 (90.0)	1 (10.0)	
Living time in Hainan (years)			<0.001	Smoking frequency			0.489
<1	231 (97.9)	5 (2.1)		Never	704 (97.4)	19 (2.6)	
1 ~ 5	227 (89.0)	28 (11.0)		≤1 time/year	19 (95.0)	1 (5.0)	
5 ~ 20	170 (84.6)	31 (15.4)		2 ~ 12 times/year	12 (92.3)	1 (7.7)	
>20	207 (62.9)	122 (37.1)		1 ~ 3 times/month	11 (91.7)	1 (8.3)	
Ethnicity			0.001	1 ~ 6 times/week	11 (100.0)	0 (0.0)	
Han	715 (82.0)	157 (18.0)		≥1 time/day	29 (93.5)	2 (6.5)	
Li	53 (96.4)	2 (3.6)		Drinking frequency			<0.001
Other	67 (71.3)	27 (28.7)		Never	412 (97.2)	12 (2.8)	
Education years			<0.001	≤1 time/year	138 (100.0)	0 (0.0)	
<6	26 (26.5)	72 (73.5)		2 ~ 12 times/year	166 (96.0)	7 (4.0)	
6 ~ 9	17 (20.0)	68 (80.0)		1 ~ 3 times/month	56 (98.2)	1 (1.8)	
9 ~ 13	756 (94.5)	44 (5.5)		1 ~ 6 times/week	8 (80.0)	2 (20.0)	
≥13	36 (94.7)	2 (5.3)		≥1 time/day	6 (75.0)	2 (25.0)	
BMI (kg/m^2^)			<0.001	Exercise frequency			0.040
<18.5	217 (93.5)	15 (6.5)		Never	26 (89.7)	3 (10.3)	
18.5 ~ 24.9	508 (86.7)	78 (13.3)		≤1 time/year	48 (96.0)	2 (4.0)	
≥25	88 (68.2)	41 (31.8)		2 ~ 12 times/year	102 (96.2)	4 (3.8)	
Pets keeping in home			0.47	1 ~ 3 times/month	239 (97.6)	6 (2.4)	
Yes	778 (81.6)	176 (18.4)		1 ~ 6 times/week	294 (98.7)	4 (1.3)	
No	57 (85.1)	10 (14.9)		≥1 time/day	77 (93.9)	5 (6.1)	
The room was damp and musty			<0.001	Vegetables intake frequency			0.409
Yes	268 (62.8)	159 (37.2)		Never	15 (93.8)	1 (6.3)	
No	567 (95.5)	27 (4.5)		≤1 time/year	11 (91.7)	1 (8.3)	
Indoor timing natural ventilation			<0.001	2 ~ 12 times/year	22 (95.7)	1 (4.3)	
Yes	650 (96.2)	26 (3.8)		1 ~ 3 times/month	41 (93.2)	3 (6.8)	
No	185 (53.6)	160 (46.4)		1 ~ 6 times/week	202 (98.1)	4 (1.9)	
Mother has asthma			0.787	≥1 time/day	495 (97.2)	14 (2.8)	
Yes	6 (85.7)	1 (14.3)		Meat intake frequency			0.834
No	829 (81.8)	185 (18.2)		Never	11 (91.7)	1 (8.3)	
Father has asthma			0.889	≤1 time/year	13 (100)	0 (0.0)	
Yes	10 (83.3)	2 (16.7)		2 ~ 12 times/year	20 (95.2)	1 (4.8)	
No	825 (81.8)	184 (18.2)		1 ~ 3 times/month	64 (97)	2 (3.0)	
Fruit intake frequency			0.006	1 ~ 6 times/week	267 (96.7)	9 (3.3)	
never	14 (87.6)	2 (12.6)		≥1 time/day	411 (97.4)	11 (2.6)	
≤1 time/year	11 (91.7)	1 (8.3)		Mattress cleaning frequency			0.014
2 ~ 12 times/year	31 (96.9)	1 (3.1)		Never	17 (94.4)	1 (5.6)	
1 ~ 3 times/month	111 (93.3)	8 (6.7)		≤1 time/year	32 (100.0)	0 (0.0)	
1 ~ 6 times/week	360 (98.9)	4 (1.1)		2 ~ 12 times/year	243 (97.2)	7 (2.8)	
≥1 time/day	256 (97.0)	8 (3.0)		1 ~ 3 times/month	387 (97.5)	10 (2.5)	
				1 ~ 6 times/week	83 (97.6)	2 (2.4)	
				≥1 time/day	24 (85.7)	4 (14.3)	

### 3.4. Multivariate analysis

**Figure 1:**
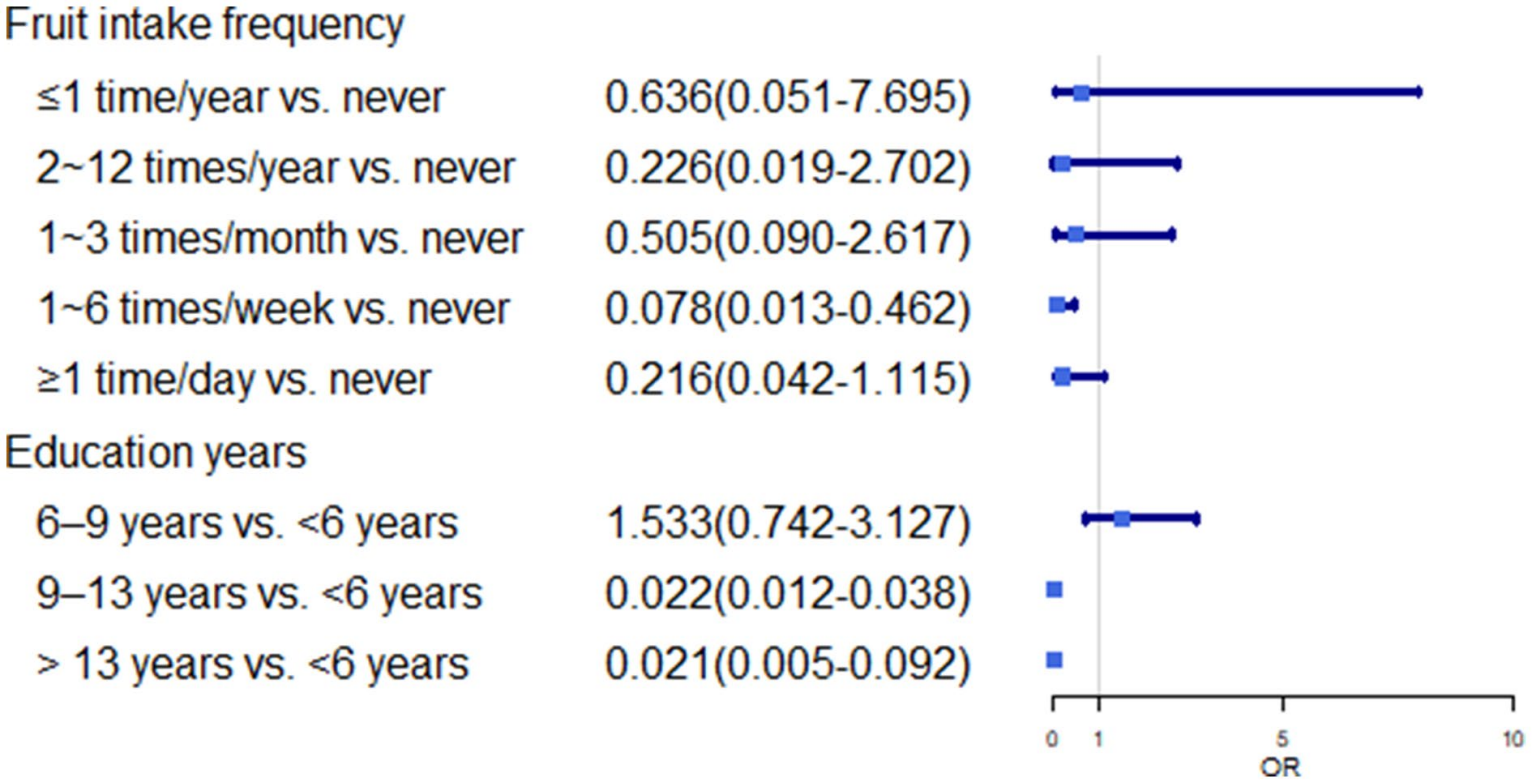
OR(95%CI) of fruit intake frequency and education years.

**Figure 2:**
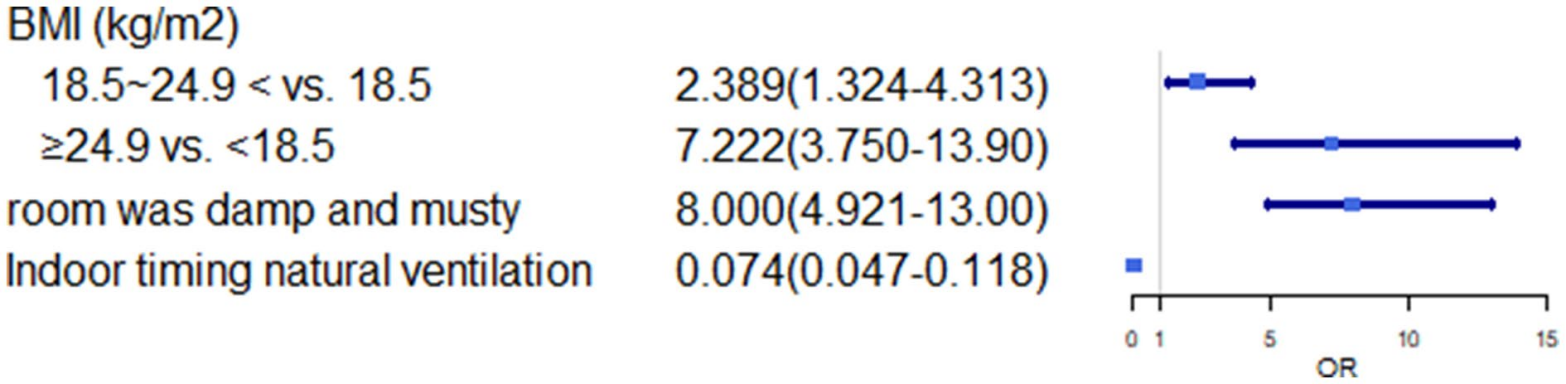
OR(95%CI) of BMI, room was damp or musty and indoor timing natural ventilation.

**Figure 3:**
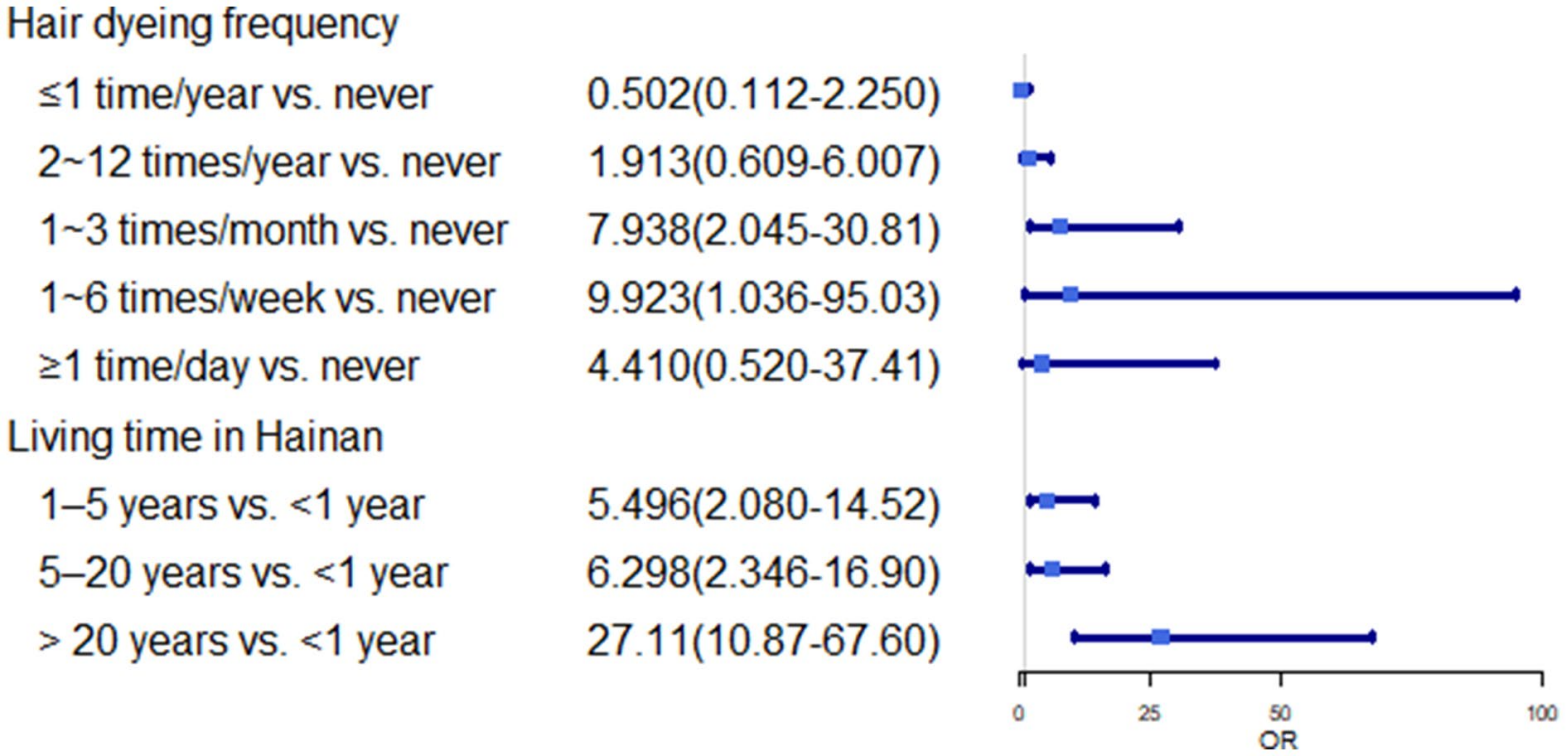
OR(95%CI) of hair dyeing frequency and living time in Hainan.

In multivariate analysis, we found some risk factors positively associated with the higher prevalence rate of asthma including longer living time in Hainan (>20 years vs. <1 year: OR, 27.111; 95%CI, 10.871 ~ 67.609), BMI (≥24.9 kg/m^2^ vs. <18.5 kg/m^2^: OR, 7.222; 95%CI, 3.750 ~ 13.907), hair dyeing (1 ~ 6 times/week vs. never: OR, 9.923; 95%CI, 1.036 ~ 95.034), the room was damp and musty (Yes vs. No: OR, 8.000; 95%CI, 4.921 ~ 13.008) (*p* < 0.05). Additionally, fruit intake (1–6 times/week vs. never: OR, 0.078; 95%CI, 0.013–0.462), years of education (>13 years vs. <6 years: OR, 0.021; 95%CI, 0.005–0.092), and indoor timing of natural ventilation (yes vs. No: OR 0.074; 95%CI, 0.047–0.118) showed a negative correlation with the prevalence rate of self-reported asthma (*p* < 0.05) (Table 4; Figures 1–3).

**Table 4:** Results of multivariate analysis (*n* = 1,021).

	*B*	S.E.	Wals	*p*	OR	Lower limit	Upper limit
The room was damp and musty (no as reference)	2.079	0.248	70.305	<0.001	8.000	4.921	13.008
Indoor timing natural ventilation (no as reference)	−2.600	0.238	119.598	<0.001	0.074	0.047	0.118
Hair dyeing frequency							
≤1 time/year vs. never	−0.688	0.765	0.810	0.368	0.502	0.112	2.250
2 ~ 12 times/year vs. never	0.649	0.584	1.234	0.267	1.913	0.609	6.007
1 ~ 3 times/month vs. never	2.072	0.692	8.963	0.003	7.938	2.045	30.815
1 ~ 6 times/week vs. never	2.295	1.153	3.963	0.047	9.923	1.036	95.034
≥1 time/day vs. never	1.484	1.091	1.851	0.174	4.410	0.520	37.411
Fruit intake frequency							
≤1 time/year vs. never	−0.452	1.289	0.123	0.726	0.636	0.051	7.965
2 ~ 12 times/year vs. never	−1.488	1.266	1.381	0.240	0.226	0.019	2.702
1 ~ 3 times/month vs. never	−0.684	0.840	0.664	0.415	0.505	0.097	2.617
1 ~ 6 times/week vs. never	−2.551	0.908	7.896	0.005	0.078	0.013	0.462
≥1 time/day vs. never	−1.531	0.837	3.349	0.067	0.216	0.042	1.115
Living time in Hainan							
1–5 years vs. <1 year	1.704	0.496	11.812	<0.001	5.496	2.080	14.522
5–20 years vs. <1 year	1.840	0.504	13.343	<0.001	6.298	2.346	16.904
> 20 years vs. <1 year	3.300	0.466	50.096	<0.001	27.111	10.871	67.609
Education years							
6–9 years (middle school) vs. <6 years	0.421	0.367	1.314	0.252	1.523	0.742	3.127
9–13 years vs.(high school). <6 years	−3.839	0.285	181.705	<0.001	0.022	0.012	0.038
> 13 years (College graduate or above) vs. <6 years	−3.887	0.765	25.831	<0.001	0.021	0.005	0.092
BMI (kg/m^2^)							
18.5 ~ 24.9 < vs. 18.5	0.871	0.301	8.352	0.004	2.389	1.324	4.313
≥24.9 vs. < 18.5	1.977	0.334	34.966	<0.001	7.222	3.750	13.907

## 4. Discussion

In this study, univariate and multivariate analyses showed that higher fruit intake frequency, years of education, and indoor timing of natural ventilation were negatively correlated with the prevalence rate of self-reported asthma (*p* < 0.05). Conversely, living in a damp and musty room, a higher frequency of hair dyeing, longer living time in Hainan, and higher BMI were positively correlated with the prevalence of self-reported asthma (*p* < 0.05). The prevalence rate of self-reported asthma in this study was 18.2%. Similarly, according to Pagán et al. (4), the five countries with the highest prevalence rates of clinical asthma were Australia (21.5%), Sweden (20.2%), the UK (18.2%), the Netherlands (15.3%), and Brazil (13.0%). This indicates that asthma is a serious public health concern. However, our estimated prevalence rate of asthma based on questionnaires was higher than the value of 5.8% reported by Wang et al. in a phone interview study of adults aged 16–65 years from 18 major cities in China (14) and also higher than that reported by Huang et al. (5) in a cross-sectional study (4.2%).

In this study, we also found that living in damp and musty rooms was a risk factor for asthma (*p* < 0.05). The tropics are regions of the Earth surrounding the equator, delimited in latitude by the Tropic of Cancer in the Northern Hemisphere and the Tropic of Capricorn in the Southern Hemisphere. Haikou and Chengmai have a tropical monsoon climate with high-temperature and high-humidity environments. The annual average temperature ranges from 23.1 to 27.0°C, and the annual average humidity is greater than 70% (15, 16). These conditions provide an ideal warm and humid environment (25–30°C with a relative humidity of 70%) for the growth and multiplication of house dust mites (HDMs) (8). Average annual temperature is the main outdoor factor that correlates with higher mite concentrations (4). Humidity is a significant limiting factor for HDMs growth and has a profound influence on reproduction (17, 18). Intensive rain and flooding induce dampness and mold proliferation in affected households, thereby affecting the quality of indoor air (19, 20). Numerous epidemiological studies have shown that the number of house dust mites in dwellings increases with increasing indoor air humidity (21). Dampness facilitates the growth of microorganisms such as bacteria and molds (22, 23). Many studies have shown that fungi and mold are associated with an increased risk of asthma (24, 25). Therefore, the climate in Hainan may have a positive impact on the production and reproduction of mold and dust mites, resulting in a higher asthma rate among local residents. Sun et al. compared the concentrations of house dust mite allergens in China and Europe and found that indoor dust mite allergen concentrations were lower in the north than in the south, both in China and Europe (26). Therefore, although the loose diagnostic criteria in this study may increase the estimated prevalence of asthma, given the unique geographical location and environment of this study and the fact that 41.8% of subjects expressed damp and musty rooms, it is possible that the prevalence of asthma in this region is indeed higher than that in other regions, and the longer living time in Hainan is associated with a higher risk of asthma. Furthermore, the prevalence of asthma in China is likely to increase rapidly due to rapid changes in the environment and lifestyle, as well as the aging population (27).

Hair dyeing was identified as a risk factor for asthma (*p* < 0.05). Para-phenylenediamine (PDD) and other members of the aromatic amine family have been extensively used as primary agents in permanent hair dyes. Presently, over two-thirds of hair dyes in the market contain PPD (28). Hairdressers are at a high risk of occupational rhinitis and asthma because, in daily work, they are frequently exposed to irritants and allergens such as persulfate and PDD in hair dyes (29, 30). *In vitro* experiments involve the use of substances, such as PDD and hydrogen peroxide, in stains to reduce the acetylation of keratinocytes. This ultimately leads to the development of allergy asthma (26). Moreover, Haltia et al. (31) showed that vegetable hair dye containing indigo powder should be considered a causative agent for occupational asthma. Therefore, reducing the frequency of hair dyeing could increase the risk of asthma.

In this study, there was a positive association between asthma and BMI (*p* < 0.05), which is consistent with the findings of Kumar (32), who reported that the prevalence of asthma was higher in overweight respondents than in those with normal weight, indicating that body weight could be a key modifiable risk factor. Similarly, A meta-analysis of several prospective studies involving more than 300,000 adults found a dose–response relationship between obesity and asthma: the odds ratio of incident asthma was 1.5 in the overweight and 1.9 in the obese groups compared with the lean group (33). Epidemiological evidence has established a relationship between obesity and asthma (33, 34). The association between asthma and BMI is particularly relevant in the context of the increasing obesity rates worldwide. Previous studies have demonstrated that obesity is correlated with the enhanced secretion of inflammatory cytokines, including IL-6, IL-1β, and TNF-α. Consequently, this leads to lower levels of systemic inflammation and higher susceptibility to worsened asthma (35). Therefore, attention should be paid to the adverse effects of obesity on asthma.

In contrast, we found a negative correlation between years of education and the prevalence rate of self-reported asthma (*p* < 0.05), which is consistent with the findings of Anto et al. (35) and Fazlollahi et al. (36). This may be because subjects with higher education levels have better health knowledge and behaviors that can help reduce the risk of asthma. Our survey also found that increased fruit intake reduced the risk of asthma (*p* < 0.05). Similarly, an overview of systematic reviews showed evidence of the beneficial effects of fresh fruits and antioxidant vitamins on asthma (37). Several studies in adults have linked high fruit and vegetable intake to a lower risk of asthma (38–41). Airways are particularly vulnerable to oxidative damage (42). In experiments, oxidants can induce many symptoms of asthma by inducing the release of proinflammatory mediators, including cytokines and chemokines (43). The fruits contain several antioxidants. Therefore, fruit intake is associated with reduced risk of asthma. Additionally, our study found that the indoor timing of natural ventilation was associated with a lower risk of asthma (*p* < 0.05). Sun et al. (26) suggested that indoor ventilation can reduce the concentration of HDMs. Vardoulakis et al. (44) believed that ventilation is a key factor affecting the indoor air quality (chemical and microbial) and moisture-related allergens (mold and dust mites) in dwellings. Therefore, indoor ventilation is often conducive to reducing the incidence of asthma allergens, thereby reducing the occurrence of asthma in the local population.

## 5. Limitations and recommendations

This study represents the first investigation of the prevalence rate of asthma and its associated influencing factors in tropical Chinese regions, and the screening factors were comprehensive. There are still shortcomings in this study. Firstly, this was a cross-sectional study, no causal inference can be made. Secondly, The research subjects in this study were mainly college students and there were fewer community residents. Therefore, the generalizability of the research conclusion is limited; it is relevant to young and middle-aged groups. Studies should appropriately expand sample populations, such as including children and adolescents, and increase the number of follow-up studies to provide sufficient information to support clinical treatment and prevention. However, this study provides important insights for subsequent analytical studies. Our study suggests that medical practitioners should educate individuals who suffer from allergies on methods to reduce the frequency of hair coloring, maintain a healthy body weight, increase fruit consumption, and regularly open windows to keep indoor humidity low. Additionally, they may consider purchasing dehumidifiers to reduce indoor humidity. These interventions aim to prevent or reduce the occurrence of allergic diseases. Then, local hospitals can educate the public about the occurrence mechanisms of allergic diseases and prevention methods, among other things to enhance the general knowledge of allergic diseases among the public.

## 6. Conclusion

The prevalence of self-reported asthma in the tropical regions of China was 18.2%. Factors such as higher frequency of hair dyeing, higher BMI, longer living in Hainan, lower frequency of fruit intake, lower years of education, a damp and musty room, and no indoor timing natural ventilation were found to increase the risk of asthma. Therefore, These findings could help raise awareness among residents of asthma and its associated risk factors. Additionally, Hainan Province is the only region in China situated in the tropics. This study offers diagnostic and treatment assistance in the field of tropical medicine, while also serving as the foundation for allergy prevention in Southeast Asia. Future studies should explore further information about the risk factors and underlying causes of asthma in tropical residents.

## Data availability statement

The raw data supporting the conclusions of this article will be made available by the authors, without undue reservation.

## Ethics statement

The studies involving humans were approved by RZ, a member of our unit, has applied for the 2021 of the National Natural Science Foundation of China. After preliminary examination by the Ethics Review Committee of Hainan Medical University. The studies were conducted in accordance with the local legislation and institutional requirements. The participants provided their written informed consent to participate in this study. Written informed consent was obtained from the individual(s) for the publication of any potentially identifiable images or data included in this article.

## Author contributions

MC: Writing – original draft, Writing – review & editing. XZ: Writing – review & editing. KZ: Conceptualization, Writing – review & editing. JG: Writing – original draft. RZ: Writing – original draft, Writing – review & editing. XW: Writing – review & editing. WC: Writing – review & editing.
